# Hepatic expression of Yin Yang 1 (YY1) is associated with the non-alcoholic fatty liver disease (NAFLD) progression in patients undergoing bariatric surgery

**DOI:** 10.1186/s12876-018-0871-2

**Published:** 2018-10-03

**Authors:** Xianwen Yuan, Jun Chen, Qi Cheng, Yinjuan Zhao, Pengzi Zhang, Xiaoyan Shao, Yan Bi, Xiaolei Shi, Yitao Ding, Xitai Sun, Bin Xue

**Affiliations:** 1 State Key Laboratory of Pharmaceutical Biotechnology, Jiangsu Key Laboratory of Molecular Medicine and School of Medicine, Nanjing University, Nanjing, Jiangsu Province China; 2 Department of Hepatobiliary Surgery, the Affiliated Drum Tower Hospital of Nanjing University Medical School, Nanjing, 210008 Jiangsu Province China; 3 Department of Pathology, the Affiliated Drum Tower Hospital of Nanjing University Medical School, Nanjing, Jiangsu Province China; 4 Collaborative Innovation Center of Sustainable Forestry in Southern China, College of Forestry, Nanjing Forestry University, Nanjing, China; 5 Department of Endocrinology, the Affiliated Drum Tower Hospital of Nanjing University Medical School, Nanjing, Jiangsu Province China; 6 State Key Laboratory of Natural Medicines, China Pharmaceutical University, Nanjing, Jiangsu Province China; 7 Liver Disease Collaborative Research Platform of Medical School of Nanjing University, 22 Hankou Road, Gulou District, Nanjing, 210093 Jiangsu Province China

**Keywords:** Yin Yang 1(YY1), Non-alcoholic fatty liver disease (NAFLD), Bariatric surgery

## Abstract

**Background:**

This study is to investigate the association between the hepatic expression of Yin Yang 1 (YY1) and the progression of non-alcoholic fatty liver disease (NAFLD) in patients undergoing bariatric surgery.

**Methods:**

Obese patients undergoing bariatric surgery were included. Liver tissues were subjected to the quantitative real-time PCR, Western blot analysis, and immunohistochemical assay, to determine the expression levels of YY1.

**Results:**

Totally 88 patients were included. According to the NAFLD activity score (NAS), these patients were divided into the control (*n* = 12), steatosis (*n* = 20), non-defining NASH (*n* = 38), and NASH (*n* = 18) groups. Significant differences in the serum glucose, insulin, ALT, AST, and HOMA-IR levels were observed among these different NAFLD groups. Hepatic YY1 expression had correlation with serum glucose, insulin, HOMA-IR, ALT, AST, triglycerides, HDL, and GGT. Immunohistochemical analysis showed that, compared with the control group, the expression levels of YY1 were significantly higher in the non-defining NASH and NASH groups. In addition, multivariate regression model showed that the serum ALT and YY1 levels were strongly associated with the NAFLD activity.

**Conclusions:**

Several factors are associated with NAFLD progression, including the expression of YY1. Our findings contribute to understanding of the pathogenesis of NAFLD.

**Trial registration:**

NCT03296605, registered on September 28, 2017.

## Background

Non-alcoholic fatty liver disease (NAFLD) represents a liver disease spectrum characterized by excessive accumulation of fat in the liver, with no alcohol abuse [1, 2]. NAFLD could be classified into the non-alcoholic fatty liver (NAFL; which is simple steatosis) and the non-alcoholic steatohepatitis (NASH) [3]. Steatosis is a benign status with mild fat deposition, which could be reversed by the lifestyle modification (such as diet and exercise) [4]. On the other hand, for NASH, in addition to the fat deposition, there would be intralobular inflammation and hepatocyte ballooning. Moreover, NASH can progress into advanced liver fibrosis, cirrhosis, and ultimate hepatocellular carcinoma (HCC) [1, 5].

NAFLD is strongly associated with obesity, dyslipidemia, diabetes, and insulin resistance, which has been therefore regarded as the hepatic manifestation of metabolic syndromes [6]. Despite massive advances in elucidating the genetic mechanism in NAFLD development, understanding of the disease pathogenesis remains incomplete [1]. Recently, the *two-hit* theory has been widely accepted to elucidate the pathogenesis of NAFL and NASH. The first *hit* refers to the accumulation of triglyceride (TG) in hepatocytes, i.e., the simple steatosis. This process is closely associated with abnormal lipid metabolism involved in central obesity and insulin resistance. The second *hit* includes mechanisms contributing to the development of inflammation and fibrosis, such as oxidative stress and mitochondrial dysfunction [7, 8].

Patients with NAFLD are always asymptomatic in clinic. The disease is often diagnosed when there is evidence for liver steatosis on imaging modality, which is associated with the metabolic syndromes, including obesity (high body mass index, BMI, and waist circumference) and diabetes (high blood glucose with hypertriglyceridemia) [5, 6]. Ultrasonography is a non-invasive method frequently used in the assessment of hepatic lipid accumulation [9, 10], so as other imaging techniques like computed tomography (CT) and nuclear magnetic resonance (NMR) [10, 11]. In addition, the blood biochemistry results could also give a hint on the diagnosis of NAFLD, such as the elevated transaminase level [12].

Recently, there are advances in the non-invasive techniques intending to assess the NASH/fibrosis level, including the NAFLD fibrosis score (NFS) [5], Fibro Meter [13, 14], and Fibro Scan [15], with, however, relatively low accuracy. Up to now, the liver biopsy is still considered to be the gold standard for the diagnosis of stages of NASH, as well as distinguishing NAFL, NASH, and liver fibrosis [16]. However, no factors against NAFLD have been elucidated to date.

Yin Yang 1 (YY1), a ubiquitous, is a multifunctional zinc-finger transcription factor from the protein family, which can work as transcriptional repressor, activator, or initiator element binding protein [17]. A myriad of potential YY1 target genes have already been identified, important for cell proliferation and differentiation process. YY1 has been shown to play an important role in regulating proliferation and apoptosis of tumor cells [18]. Moreover, YY1 promotes the triglyceride accumulation in the adipocytes via repressing Chop10 transcription, implying its potential role in the development of obesity [19]. Furthermore, YY1 has also been found to be able to repress the genes associated with the insulin/insulin-like growth factor (IGF) signaling pathway, such as IGF1–2, IRS1–2, and Akt1–3 in skeletal muscles [20]. A recent study has also found that YY1 might be related to the body weight, glucose level, and cholesterol or free fatty acid level [21]. In addition, compared with control subjects, the YY1 levels are significantly down-regulated in the liver tissues in NAFLD patients [22]. However, the association between the YY1 expression and the NAFLD progression has not completely elucidated.

In this study, the obese patients undergoing bariatric surgery were divided into four groups according to the liver pathogenesis. The mRNA and protein expression levels of YY1 were determined, and the association between the YY1 expression and the NAFLD progression was investigated.

## Methods

### Study subjects

This study was approved by the Ethics Committee of the Affiliated Drum Tower Hospital of the Medical School of Nanjing University (Permit Number: 2017–030-02). This study was registered in International Clinical Trial Registry Platform (ICTRP), with the clinical trial number NCT03296605. Patients were selected from a cohort undergoing laparoscopic Roux-en-Y gastric bypass surgery at the Department of Hepatobiliary Surgery of the Affiliated Drum Tower Hospital of the Medical School of Nanjing University. Exclusion criteria were included the patients with evidence for viral hepatitis, hemochromatosis, or alcohol consumption (> 20 g/d for females and > 30 g/d for males) [23]. The participants were recruited from April 2017 to February 2018. Written informed consent was obtained from all subjects.

### Data collection

Liver tissue samples were obtained during surgery. One half was put into lipid nitrogen and stored at − 80 °C; and the other half was fixed by 10% formaldehyde, embedded in paraffin, and subjected to the hematoxylin-eosin (H&E) staining. Specimen was stored in the Nanjing Multicenter Biobank, the Biobank of Nanjing Drum Tower Hospital, and the Affiliated Hospital of Nanjing University Medical School. We conducted this study from February 2018. We had access to information that could identify individual participants during or after data collection. Histological characteristics were determined according to the Kleiner scoring system [24]. Steatosis was assessed and scored in a scale of 0–3, inflammation grades of 0–3, and hepatocellular ballooning of 0–2. These histopathological features were used to estimate the NAFLD activity score (NAS). These subjects were classified into the control (without steatosis), hepatic steatosis (NAS of 1–2), non-defining NASH (NAS of 3–4), and NASH (NAS of ≥5) groups [25]. Fibrosis was staged in based on the grades of 0–4. For biochemical measurement, blood samples were taken after an overnight (10-h) fast. Samples were analyzed and tested for the liver function, insulin level, C-reactive protein level, glucose level, and lipid panels (including total cholesterol, LDL, HDL, and triglycerides). Insulin activity was determined by the homeostatic model assessment for insulin resistance (HOMA-IR) index [26, 27].

### Quantitative real-time PCR

Total RNA was extracted from the liver tissue using Trizol (Invitrogen, Carlsbad, CA, USA). RNA (500 ng) was used for cDNA synthesis using random primers and Primescriptreverse transcriptase (Takara, Dalian, Liaoning, China). Quantitative real-time PCR was carried out using the SYBR Green qPCR kit (Takara), on a fluorescent temperature cycler. Primer sequences were as follows: YY1, forward 5′-ACGGCTTCGAGGATCAGATTC-3′ and reverse 5′-TGACCAGCGTTTGTTCAATGT-3′; and GAPDH, forward 5′-TGACTTCAACAGCGACACCCA-3′ and reverse5′-CACCCTGTTGCTGTAGCCAAA-3′. Reaction conditions were set as: 95 °C for 30 s, followed by 40 cycles of 95 °C for 5 s and 60 °C for 34 s. Target gene expression was calculated with semi-quantitative method. GAPDH was used as internal reference.

### Western blot analysis

Tissues were lysed with the RIPA buffer containing phosphatase inhibitors. The protein concentration was determined using the BCA method (Pierce, Rockford, IL, USA). Totally 24 mg protein was separated on 10% SDS-PAGE, and then electronically transferred onto a PVDF membrane. After blocking with 3% BSA in 10 mM Tris-HCl (pH 7.4) containing 0.05% Tween-20, the membrane was incubated with the mouse anti-YY1 (Abcam, Cambridge, MA, USA) and anti-β-actin primary antibody (1:1000 dilution; Key GEN Bio TECH, Nanjing, Jiangsu, China) at 4 °C overnight. After washing, the membrane was incubated with the peroxidase-conjugated secondary antibody (Santa Cruz, Santa Cruz, CA, USA), and developed in the Super Signal West Pico Chemiluminescent Substrate (Pierce). The protein was visualized and quantified with the Imagine J software.

### Immunohistochemistry analysis

Formalin-fixed liver tissue samples were subjected to the immunohistochemistry analysis. Briefly, liver sections were deparaffinized and treated by citrate, and then blocked with Immuno Detector Peroxidase Blocker (Bios SB, Santa Barbara, CA, USA). Sections were incubated with the rabbit anti-YY1 primary antibody (1:250dilution; Abcam, Cambridge, MA, USA) at 4 °C overnight. Liver sections were then treated with peroxidase-conjugated secondary antibody (Santa Cruz) and DAB chromogen. Then the samples were counterstained with hematoxylin, and observed under light microscope.

### Statistical analysis

Data were expressed as mean ± SD. Statistical analysis was performed using the SPSS 19.0 software (SPSS Inc., Chicago, IL, USA). Group comparison of numeric variables was performed using the ANOVA or Kruskal-Wallis test, depending on the variables’ distribution. The χ^2^test was used for comparison of nominal categorical variables. Correlation analysis was conducted with the Spearman’s test. Multivariate logistic regression model was used to identify the significant clinical and metabolic factors that predicted the NAFLD absence, after adjusting for other factors such as BMI.

## Results

### Clinical characteristics of study population

Totally 88 patients were included in this study. Clinical and biochemical characteristics of these patients were shown in Table 1. In these subjects, there were 12 cases without steatosis (13.6%), 20 cases of steatosis (22.7%), 38 cases of non-defining NASH (43.2%), and 18 cases of NASH (20.5%). Our results showed that the glucose levels were significantly changed along with the NAFLD progression. Significant differences were observed between the control and non-defining NASH groups, the control and NASH groups, and the non-defining NASH and NASH groups (all *P* < 0.05). Moreover, the insulin level was significantly changed along with the NAFLD progression. Significant differences were observed between the control and non-defining NASH groups, the control and NASH groups, the steatosis and non-defining NASH groups, and then steatosis and NASH groups (all *P* < 0.05). Furthermore, the HOMA-IR was significantly changed along with the NAFLD progression. Significant differences were observed between all these groups (*P* < 0.05), except for the non-defining NASH and NASH groups. In addition, the ALT levels were significantly changed along with the NAFLD progression. Significant differences were observed between all the groups (*P* < 0.05), except for the control and steatosis groups, and the steatosis and non-defining NASH groups. Besides, the AST levels were significantly changed along with the NAFLD progression. Significant differences were observed between all the groups (all *P* < 0.05), except for the control and steatosis groups, and the control and non-defining NASH groups. However, no significant differences were observed in other biochemical parameters between these groups. Taken together, these results suggest significantly different serum ALT, AST, glucose, insulin, and HOMA-IR levels at different NAFLD stages.

**Table 1:** Anthropometric and biochemical parameters of the study subjects

	Control (*n* = 12)	Steatosis (*n* = 20)	Non-defining NASH (*n* = 38)	NASH (*n* = 18)	*P*
Age (years)	33.4 ± 12.9	39.1 ± 12.1	34.2 ± 10.4	34.2 ± 13.1	NS
BMI (kg/m^2^)	38.1 ± 8.5	37.7 ± 5.02	40.4 ± 7.3	39.4 ± 5.9	NS
Glucose (mmol/L)	4.9 (4.6–5.3)	5.5 (4.8–7.3)	5.5 (5.2–6.5)	6.6 (5.7–8.8)	< 0.05^#^, ^∆^, ^&^, ^╪^
Insulin (μIU/mL)	15.3 (12.9–23.1)	19.6 (12.9–31.4)	26.5 (19.6–41.5)	33.4 (20.0–50.8)	< 0.05^#^, ^∆^, ^§^, ^&^
HOMA-IR	3.6 (3.1–4.4)	4.9 (3.9–8.2)	7.3 (5.2–10.6)	11.5 (5.8–16.9)	< 0.05*, ^#^, ^∆^, ^§^, ^&^
C reactive protein (mg/L)	4.95 (2.75–6.85)	5.9 (4.1–7.4)	6.7 (5.5–11.3)	6.8 (5.1–10.3)	NS
Triglycerides (mmol/L)	1.59 (0.96–2.45)	1.43 (1.18–2.04)	1.90 (1.23–2.34)	1.95 (1.52–6.29)	NS
Total Cholesterol (mmol/L)	4.7 ± 0.9	4.8 ± 0.9	4.8 ± 0.6	5.4 ± 1.0	NS
HDL-C (mmol/L)	1.05 (0.96–1.50)	1.12 (0.99–1.27)	1.01 (0.83–1.10)	0.92 (0.75–1.02)	NS
LDL-C (mmol/L)	3.09 (1.83–3.36)	2.94 (2.15–3.12)	2.71 (2.16–3.35)	2.53 (2.29–3.51)	NS
ALT (IU/mL)	19.4 (14.0–28.6)	25.2 (21.0–37.5)	35.8 (27.3–60.2)	76.5 (50.3–128.8)	< 0.05^#^, ^∆^, ^&^, ^╪^
AST (IU/mL)	19.5 (15.0–25.4)	20.2 (14.7–25.9)	24.1 (18.5–35.2)	50.5 (29.2–90.7)	< 0.05^∆^, ^§^, ^&^, ^╪^
GGT (IU/mL)	35.4 (21.6–53.3)	39.5 (20.2–66.5)	43.5 (26.5–64.0)	55.8 (37.5–90.5)	NS

Note: *BMI* body mass index, *HOMA-IR* homeostasis model assessment for insulin resistance, *HDL-C* high-density lipoprotein cholesterol, *LDL-C* low-density lipoprotein cholesterol, *ALT* alanine transaminase, *AST* aspartate transaminase, and *GGT* gamma glutamyltranspeptidase. ^*^, *P* < 0.05 between the control and steatosis groups; ^#^, *P* < 0.05 between the control and non-defining NASH groups; ^∆^, *P* < 0.05 between the control and NASH groups; ^§^, *P* < 0.05 between the steatosis and non-defining NASH groups; ^&^, *P* < 0.05 between the steatosis and NASH groups; ^╪^, *P* < 0.05 between the non-defining NASH and NASH groups; and NS, none significance

### Pathological characteristics of study population

**Fig. 1:**
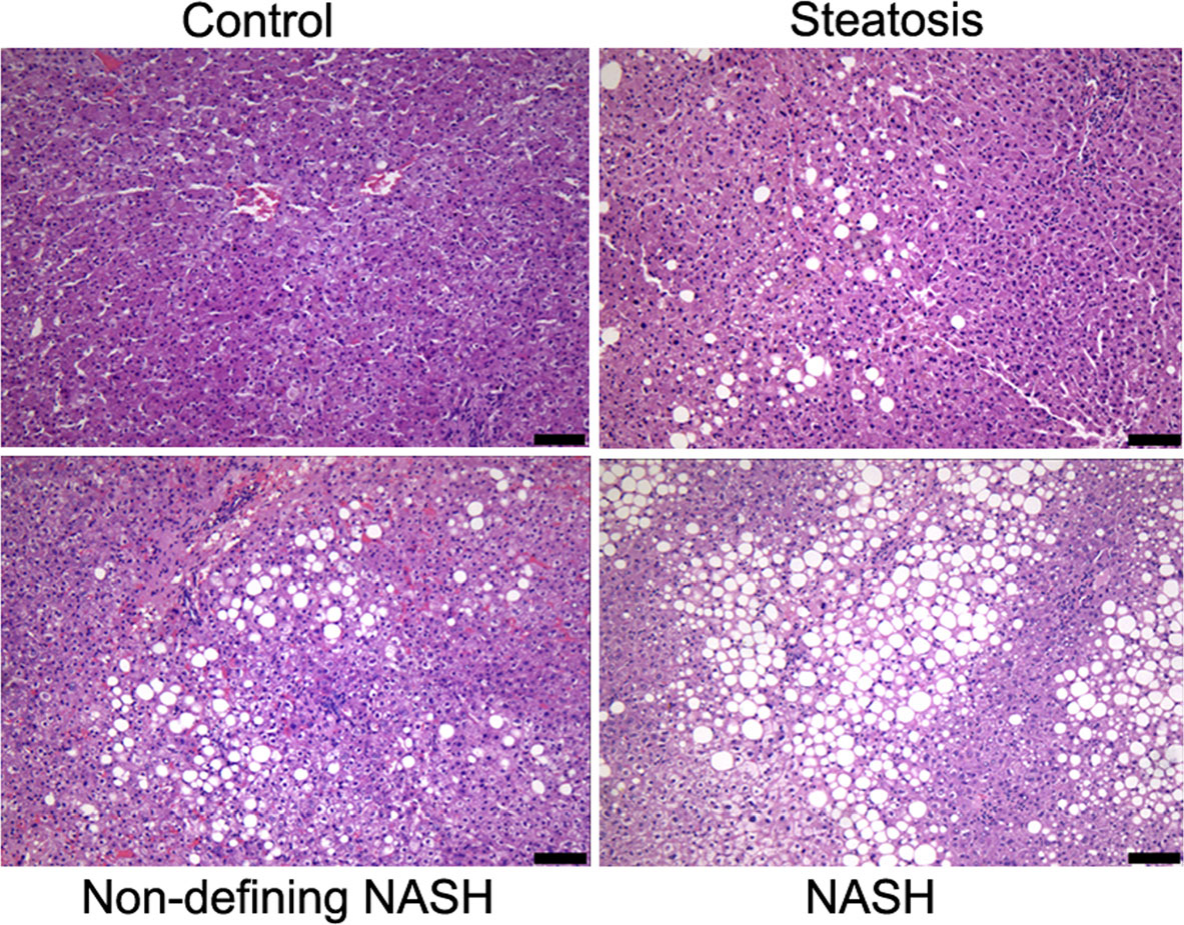
H&E staining for NAFLD at different stages. Representative pictures from the H&E staining of the control, steatosis, non-defining NASH, and NASH groups, respectively. Scale bar, 100 μm

Before surgery, the included patients subjected to the liver spy, and the pathological sections were evaluated and analyzed by pathologists. As shown in Table 2, the results of liver histology showed that, there were 20 case of grade 0 steatosis, 32 cases of grade 1 steatosis, 17 cases of grade 2 steatosis, and 19 cases of grade 3steatosis. For the fibrosis score, there were 25, 46, 14, 2, and 1 cases of scores 0–4, respectively. Moreover, there were 33 cases with lobular inflammation score 0, 47 cases with score 1, and only 8 cases with score 2. Furthermore, there were 22 cases with hepatocyte ballooning score 0, 45 cases with score 1, and 21 cases with score 2. The NAS activity was based on the above scores, and these patients could be divided into four groups accordingly. A representative slice was shown in Fig. 1. Taken together, these results suggest that, NAFLD is very common in obese population.

**Table 2:** Histological characteristics of liver in the study subjects

	Patients (*n* = 88)
	n (%)
Steatosis grade	
0	20 (22.7)
1	32 (36.4)
2	17 (19.3)
3	19 (21.6)
Fibrosis stage	
0	25 (28.4)
1	46 (52.3)
2	14 (15.9)
3	2 (2.3)
4	1 (1.1)
Lobular inflammation	
0	33 (37.5)
1	47 (53.4)
2	8 (9.1)
3	0 (0)
Hepatocyte ballooning	
0	22 (25.0)
1	45 (51.1)
2	21 (23.9)
NASH activity score (NAS)	
0	12 (13.6)
1–2	20 (22.7)
3–4	38 (43.2)
5–8	18 (20.5)

### YY1 expression and association with exact NAFLD progression

**Fig. 2:**
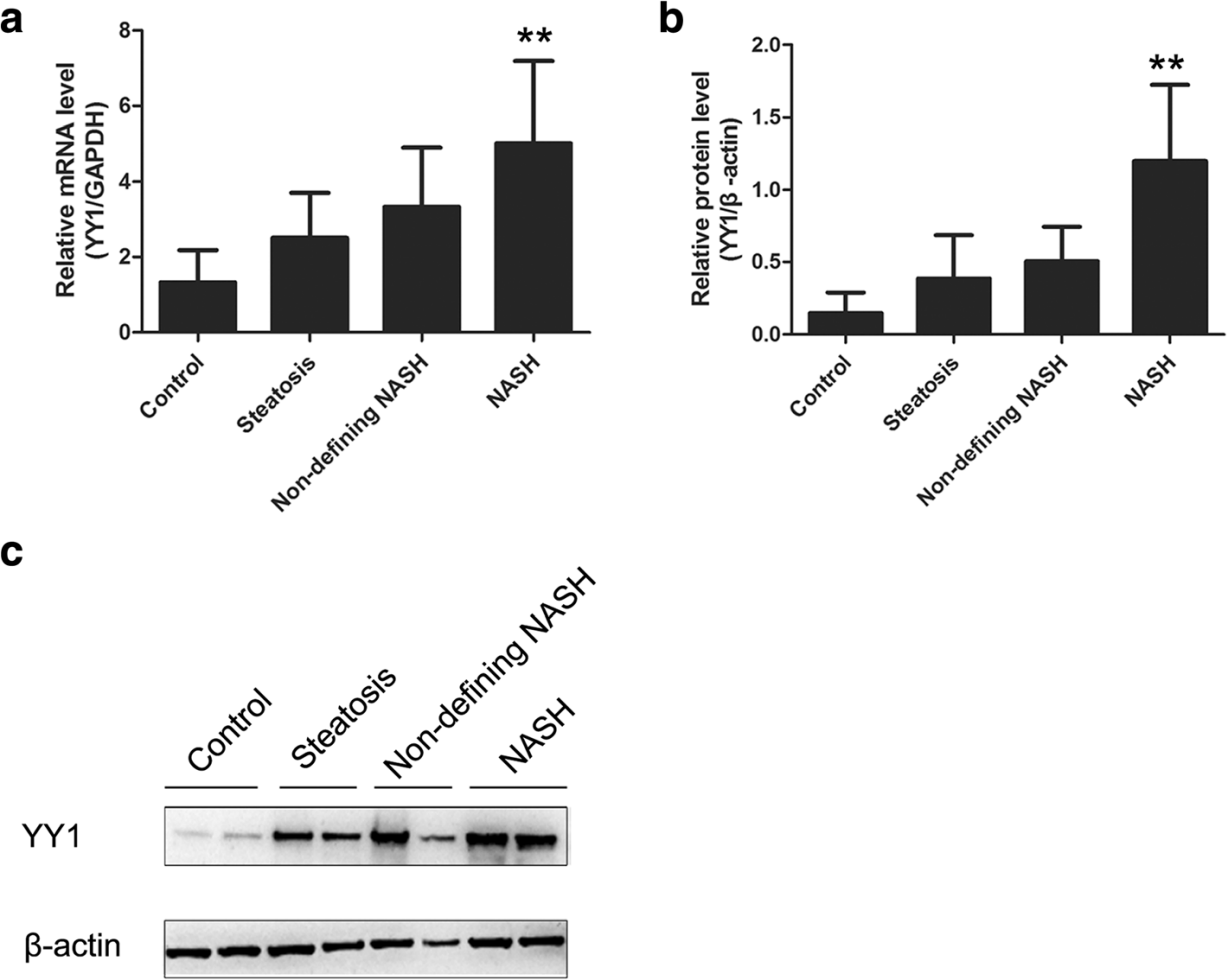
YY1 expression for NAFLD at different stages. **a** The YY1 mRNA levels in the control, steatosis, non-defining NASH, and NASH groups were detected with the quantitative real-time PCR. **p < 0.01 compared with control group. **b** The YY1 protein levels in the control, steatosis, non-defining NASH, and NASH groups were detected with western blot analysis, respectively. **p < 0.01 compared with control group. **c** Representative image of western blot in different groups

**Fig. 3:**
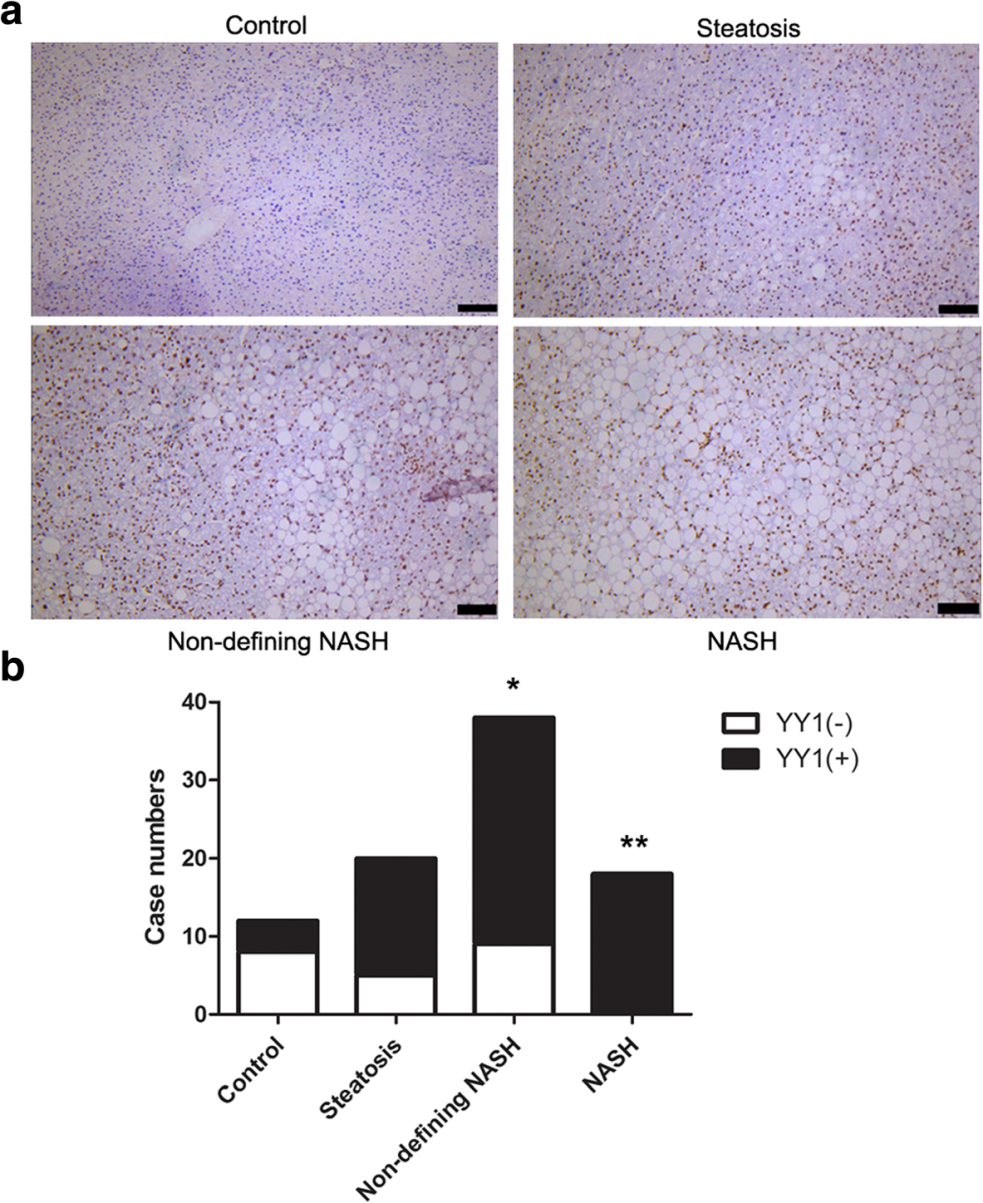
Immunohistochemistry detection of YY1 for NAFLD at different stages (**a**) Immunohistochemistry analysis was performed to detect the expression of YY1 in the control, steatosis, non-defining NASH, and NASH groups.Scale bar, 100 μm. **b** The cases positive or negative for YY1 expression were analyzed. *p < 0.05 compared with control group, **p < 0.01 compared with control group

Although YY1 expression is high in the NAFLD patients, the association between YY1 and the exact NAFLD progression has not yet been explored. To investigate the expression of YY1 at different NAFLD stages, the quantitative real-time PCR and Western blot analysis were performed. Our results showed that, compared with the control group, the mRNA and protein level of YY1 in the NASH groups was significantly elevated (Fig. 2). As shown in the Table 3, the YY1 mRNA level was significantly correlated with the serum ALT (*r* = 0.339, *P* = 0.001), AST (*r* = 0.216, *P* = 0.043), glucose (*r* = 0.274, *P* = 0.01), insulin (*r* = 0.313, *P* = 0.003), and HOMA-IR (*r* = 0.355, P = 0.001) levels. As shown in Table 4, statistical analysis indicated that the YY1 protein expression level was significantly correlated with the serum ALT (*r* = 0.459, *P* = 0.001), glucose (*r* = 0.438, *P* = 0.001), insulin (*r* = 0.369, *P* = 0.001), and HOMA-IR (*r* = 0.463, P = 0.001) levels. On the other hand, for the hepatic sections, our results showed that the YY1 expression was associated with the NAFLD progression. In the control group, there were 8 patients negative for YY1 and 4 patients positive for YY1. In the steatosis group, there were 5 patients negative for YY1 and 15 patients positive for YY1. In non-defining NASH group, there were patients negative for YY1 and 28 patients positive for YY1. In the NASH group, all the 18 patients were positive for YY1. Compared with the control group, the expression of YY1 was significantly higher in the NASH and non-defining NASH groups (Fig. 3). Taken together, these results suggest that, YY1 expression levels are not the same at different NAFLD stages.

**Table 3:** Correlation between hepatic YY1 mRNA levels and biochemical parameters

	Association with YY1 mRNA levels	
	Correlation coefficient	*P*
Age (years)	0.2	0.578
BMI (kg/m^2^)	0.1	0.515
Glucose (mmol/L)	0.3	0.010
Insulin (μIU/mL)	0.2	0.003
HOMA-IR	0.4	0.001
C reactive protein (mg/L)	0.3	0.165
Triglycerides (mmol/L)	0.1	0.977
Total cholesterol (mmol/L)	0.3	0.768
HDL (mmol/L)	0.1	0.965
LDL (mmol/L)	0.1	0.841
ALT (IU/mL)	0.1	0.001
AST (IU/mL)	0.1	0.043
GGT (IU/mL)	0.3	0.808

**Table 4:** Correlation between hepatic YY1 protein levels and biochemical parameters

	Association with YY1 protein levels	
	Correlation coefficient	*P*
Age (years)	0.2	0.519
BMI (kg/m^2^)	0.4	0.080
Glucose (mmol/L)	0.3	0.001
Insulin (μIU/mL)	0.5	0.001
HOMA-IR	0.3	0.001
C reactive protein (mg/L)	0.2	0.106
Triglycerides (mmol/L)	0.1	0.043
Total cholesterol (mmol/L)	0.1	0.273
HDL (mmol/L)	0.2	0.009
LDL (mmol/L)	0.2	0.423
ALT (IU/mL)	0.3	0.001
AST (IU/mL)	0.3	0.001
GGT (IU/mL)	0.2	0.049

### NAFLD activity predicting factors

To investigate the relationship between NAFLD and YY1, multivariable linear regression model was used. Our results showed that glucose, insulin, HOMA-IR, ALT, and AST were associated with the NAFLD progression. Therefore, a multivariate linear regression model was constructed to predict NAFLD activity score (NAS). According to this model, the ALT and hepatic YY1 protein content were independent predictive factors associated with NAS Table 5. The following equation was obtained based on these results, i.e., NAS activity = 0.181 (Glucose) + 0.023 (Insulin) - 0.024 (HOMA-IR) + 0.018 (ALT) - 0.013 (AST) + 2.259 (YY1) - 0.242. Taken together, these results suggest that, the NAS activity is significantly associated with the serum ALT level and hepatic YY1 protein level.

**Table 5:** Multivariate regression model predicting NAS

	B	SE	*P*
Constant	−0.242	0.809	0.765
Glucose	0.181	0.119	0.131
Insulin	0.023	0.030	0.446
HOMA-IR	−0.024	0.093	0.800
ALT	0.018	0.006	0.007
AST	−0.013	0.012	0.270
Hepatic YY1 protein	2.259	0.317	0.000

## Discussion

In the present study, the factors associated with normal liver histology in patients with obesity were identified and investigated. Our findings identifying the protective factors could help guide the NAFLD screening among the patients with high risk, as well as further understand the disease pathogenesis. Patients undergoing weight-loss surgery offered insights into the unique patient subset. This cohort allowed for the identification of the protective factors against the development of NAFLD confirmed by histology in the high risk group. Our results showed thatYY1 was associated with the NAFLD progression. Furthermore, YY1 had strong association with glucose, insulin, HOMA-IR, ALT, and AST. These findings suggest that besides NAFLD, YY1 is also associated with the hepatic metabolism.

Recently, it has been shown that the hepatic YY1 expression level is increased in the diabetic rats [28]. Moreover, YY1 promotes the hepatosteatosis and insulin resistance, mainly via FXR, in the animal model [22]. FXR is a metabolic nuclear receptor, abundantly expressed in the liver, intestine, and kidney, which has been first identified as a key regulator in the cholesterol and bile acid homeostasis [29]. Moreover, FXR is also a major transcriptional factor participating in the regulation of the glucose and lipid metabolism in liver. In line with this, our results showed that YY1 might influence the liver metabolism. Moreover, YY1 and ALT were most important factors to predict the NAFLD activity, further supporting the important interaction between the YY1 and NAFLD progression. The more severe NAFLD was, the higher the YY1 expression level would be. Taken together, these results suggest that the hepatic YY1 expression is an important factor involved in the progression of NAFLD. This study has important clinical significance for diagnose and treatment of NAFLD. And combination of YY1 and NAS scores can serve as a more accurate diagnostic indicator for NAFLD.

There are also limitations about this study. The data was derived from the cohort of patient undergoing bariatric surgery. However, there is need for confirmation in an additional cohort which including patients selected at daily routine in a hepatological setting for NASH, outside the setting for bariatric surgery, and it should be evaluated more broadly in healthy people. Actually, the data is difficult to collect because healthy people and patients without NASH usually reject invasive testing especially in China, so it’s not feasible to confirm our conclusion in another cohort in this study. Of course, further in-depth studies are still needed to investigate the correlation between YY1 and NAFLD progression in broader populations in the future.

## Conclusion

In conclusion, this study identified factors associated with the development of NAFLD in obese patients undergoing bariatric surgery. Our results showed that YY1 had strong association with the NAFLD progression, which contributed to understanding the underlying mechanisms of NAFLD. This is the first study reporting the association between the hepatic YY1 expression and NAFLD at different stages. Our findings suggest that YY1 may be a promising therapeutic target for fatty liver diseases and related metabolic disorders in clinic.

## Abbreviations

ALT: Alanine transaminase
AST: Aspartate transaminase
CT: Computed tomography
H&E: Hematoxylin-eosin
HCC: Hepatocellular carcinoma
HOMA-IR: Homeostatic model assessment for insulin resistance
ICTRP: International Clinical Trial Registry Platform
IGF: Insulin/insulin-like growth factor
NAFL: Non-alcoholic fatty liver
NAFLD: Non-alcoholic fatty liver disease
NAS: NAFLD activity score
NASH: Non-alcoholic steatohepatitis
NFS: NAFLD fibrosis score
NMR: Nuclear magnetic resonance
TG: Triglyceride
YY1: Yin Yang 1
